# Effects of lithium chloride on queen egg‐laying performance and worker honey bee behavior

**DOI:** 10.1002/ps.70253

**Published:** 2025-09-27

**Authors:** Sevin Sedat, Jakob Avi Shimshoni, Afik Ohad, Zarhin Shlomo, Hagai Yehoshua Shpigler

**Affiliations:** ^1^ Department of Pharmacology and Toxicology Faculty of Veterinary Medicine, Ankara University Ankara Turkey; ^2^ Department of Food Science, Institute for Postharvest and Food Sciences Agricultural Research Organization, Volcani Center Rishon LeZion Israel; ^3^ Agricultural Extension Service Ministry of Agriculture and rural development Bet Dagan Israel; ^4^ Department of Entomology Agricultural Research Organization, Volcani Center Rishon LeZion Israel

**Keywords:** LiCl, honey bees behavior, aggression, oviposition, brood care

## Abstract

**BACKGROUND:**

Lithium chloride (LiCl) has recently emerged as a potential treatment for *Varroa* in honey bee colonies, yet its effects on bee behavior remain poorly understood. This study investigates, for the first time, the impact of oral exposure to chronic 50 mM LiCl—administered via candy over 7–10 days—on three key behavioral traits: queen oviposition, worker brood care, and worker aggression. Laboratory assays were conducted using caged bees with *ad libitum* access to LiCl‐enriched or control candy.

**RESULTS:**

Egg‐laying rates did not differ significantly between LiCl‐treated and control groups. Worker bees exposed to LiCl showed a significant increase in mite mortality. Brood care behavior, assessed using a 4‐day‐old queen larva, was unaffected in terms of nursing frequency and duration. However, LiCl‐treated workers exhibited a significant reduction in aggressive behaviors compared to controls.

**CONCLUSIONS:**

LiCl did not adversely influence queen egg laying or workers' nursing behavior, reinforcing previous findings suggesting negligible long‐term risks to essential colony maintenance activities such as queen reproductive performance and nursing behavior. The reduced aggression observation is consistent with reports from other species, suggesting a conserved modulatory role of Li on aggression across the animal kingdom. From an applied perspective, these results imply that LiCl can be used as *Varroa* control without compromise on colony reproduction and brood care. Furthermore, LiCl could potentially facilitate the management of aggressive colonies, thereby reducing stress for both the bees and their handlers. © 2025 The Author(s). *Pest Management Science* published by John Wiley & Sons Ltd on behalf of Society of Chemical Industry.

## Graphical abstract

Chronic oral lithium chloride (LiCl) exposure effectively increases Varroa mite mortality without impairing queen egg laying or brood care behaviors, while significantly reducing worker bee aggression, suggesting its potential for safe Varroa management and mitigating colony aggression.

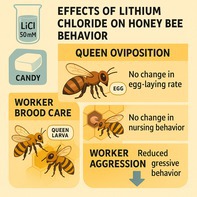

## 1 INTRODUCTION

Honey bees (*Apis mellifera*) are essential pollinators that sustain a large fraction of global agriculture, ensuring the production of fruits, vegetables, and other important crops.^1^ Among the various threats to bee health, the ectoparasitic mite *Varroa destructor* has emerged as the most severe challenge, exerting a profound negative impact on colony survival and productivity.^2^ Varroa mites feed on developing bee brood and adult bees, compromising their immune systems and vectoring a range of debilitating viruses, most notably Deformed Wing Virus.^2^ As infestations intensify, colonies experience severe brood loss, reduced lifespan of adult workers, and diminished overall colony vigor, often culminating in total collapse.^2^ The severity of the *Varroa* problem has accelerated the development of diverse control measures, including chemical (acaricides), biological, and cultural strategies.^3^ Chemical acaricides, such as pyrethroids (e.g., fluvalinate), organophosphates (e.g., coumaphos), and formamidine compounds (e.g., amitraz), have been widely used for *Varroa* control.^3^ However, these interventions are not without drawbacks. Over‐reliance on chemical treatments has led to the emergence of acaricide‐resistant *Varroa* populations, rendering once‐effective treatments less reliable over time.^2^, ^3^ Recently, a severe loss of 62% of honey bee colonies was recorded during 2024 in the United States, with Varroa populations resistant to the widely used acaricide amitraz suspected as the primary cause of this dramatic decline in survival.^4^ Moreover, residues of these chemicals can accumulate in hive products such as honey, wax, and pollen, raising concerns regarding food safety, environmental contamination, and potential sublethal effects on bees, including impaired foraging performance as well as learning and memory.^5^, ^6^, ^7^ Organic acids (e.g., formic and oxalic acid) and essential oil‐based treatments (e.g., thymol) offer more ‘natural’ alternatives, but their efficacy can vary with temperature, application method, and colony conditions, and they may still pose risks if applied improperly.^2^, ^3^ Non‐chemical methods, such as breeding for *Varroa*‐resistant bee strains, drone brood removal, and brood interruption techniques, show promise but often require substantial labor, time, and meticulous management, making their widespread implementation challenging.^8^ Together, these limitations underscore the urgent need for novel, sustainable, and integrated *Varroa* control strategies that minimize harm to both bees and their environment.

In recent years, lithium (Li) salts and particularly lithium chloride (LiCl) have gained attention as promising candidates for managing *Varroa* infestations.^9^, ^10^, ^11^, ^12^, ^13^, ^14^ This finding was a result of a side effect of a different strategy for *Varroa* management using dsRNA.^14^ Li salts occur naturally in various geological settings, including mineral springs, brines, and certain rock formations, resulting in their ubiquitous presence in soil, groundwater, and plant tissues at trace levels.^15^ Environmental concentrations can fluctuate widely, influenced by factors such as regional geology, industrial activity, and agricultural practices, yet they generally remain low enough to pose negligible risk to terrestrial and aquatic organisms under normal circumstances.^15^ Li, in various salt forms, has also been used extensively in human medicine, particularly in psychiatry, where Li carbonate and Li citrate are employed for the long‐term management of bipolar disorder at daily dosages ranging between 0.2–1.2 g/day.^16^ These medical uses underscore Li's potential to alter physiological pathways, as its salts can modulate neurological functions and behavior at carefully controlled therapeutic doses.^16^

Recently, in the context of apiculture, Li salts, particularly LiCl and Li format have shown potential in controlling *Varroa* due to their relatively high acaricidal activity at carefully chosen concentrations.^9^, ^10^, ^11^, ^12^, ^13^, ^14^ The conclusions of the aforementioned studies clearly indicated that proper dosing as well as application frequency, exposure duration and timing are critical for effective *Varroa* suppression, and honey bee safety. Toxicity to bees and brood is a primary concern when using LiCl.^10^, ^11^, ^17^ Adult bees generally tolerate LiCl at moderate levels, yet younger developmental stages—larvae and pupae—appear more sensitive.^10^, ^11^, ^17^ High‐dose or prolonged exposures have been recently reported to lead to brood mortality, reduced brood viability, and sublethal behavioral changes in adult workers.^9^, ^10^, ^11^, ^13^

Understanding the impact of LiCl on essential behavioral traits in honey bees is of critical importance, given its emerging role as a treatment against *Varroa*. While LiCl demonstrates promising varroacidal efficacy, its potential effects on fundamental social behaviors crucial for colony maintenance and survival remain largely unexplored. Queen oviposition ensures the continuous renewal of the colony's workforce, while worker brood care safeguards the development of future generations, directly influencing colony growth and resilience.^18^ Additionally, worker aggression toward intruders serves as a primary defense mechanism, protecting the colony from external threats such as robbing bees and other predators.^19^ Any impairment of these behaviors could compromise colony homeostasis, leading to weakened social organization, reduced productivity, and increased vulnerability to environmental stressors. Despite LiCl's therapeutic potential, there is a significant gap in our knowledge regarding its influence on these vital behavioral parameters. Addressing this gap is essential to fully evaluate the suitability and safety of LiCl in apicultural practice, ensuring that treatments aimed at controlling *Varroa* infestations do not inadvertently undermine the social integrity and survival of honey bee colonies.

Accordingly, this study investigates for the first time the influence of LiCl on queen egg‐laying activity and honey bee workers nursing and aggression behavior. By exploring these broader parameters, our research moves beyond basic efficacy of LiCl as an anti‐*Varroa* treatment, offering deeper insight into the ecological and physiological significance of LiCl treatment for entire honey bee colonies.

## 2 MATERIALS AND METHODS

### 2.1 Reagents

LiCl was purchased from Sigma‐Aldrich (St. Louis, MO, USA) at a purity grade above 99%. Food grade sucrose was purchased from local grocery stores. Honey for the preparation of the candy formulations was obtained from our in‐house beehives. The honey was tested for the presence of Li as well as in Israel approved acaricide residues (amitraz, coumaphos) at The Scientific Service Core Facility, The Faculty of Agricultural, Food, and Environmental Quality Sciences, The Hebrew University, Rehovot, Israel (see below).

### 2.2 Li quantification in honey by inductively coupled plasma mass spectrometry (ICP‐MS)

Prior to analysis, all plastic and glassware were cleaned with 5% HNO_3_ (prepared from 65% concentrated HNO_3_, Merck) and Milli‐Q deionized water (Darmstadt, Germany). Teflon digestion vessels were cleaned with detergent, rinsed thoroughly with deionized water, and subjected to a microwave‐assisted acid‐cleaning cycle using 5 mL of 65% HNO_3_ and deionized water, followed by final rinsing with deionized water. For analysis, 500 mg of honey (collected from the in‐house beehive used for candy preparation) was accurately weighed (Nimbus NBL Series, Adam Equipment Ltd, UK). Samples were digested in Teflon vessels with 5 mL of 65% HNO_3_ and 1 mL of 30% H_2_O_2_ (Merck, Germany) using a microwave digestion system (‘Ethos Easy,’ Milestone, Italy), with heating from 80 to 210 °C to ensure complete dissolution. Digested samples were cooled to room temperature, transferred to polypropylene flasks, and diluted to 20 mL with deionized water. Li quantification was performed by ICP‐MS (‘Plasma Quant MS Elite,’ Analytik Jena, Germany), targeting the ^7^Li isotope. The instrument underwent daily calibration and automatic optimization. An internal standard (^103^Rh at 5 μg/L) was continuously introduced into all solutions via a Y‐connector to ensure analytical precision. The method achieved a limit of quantification (LOQ) of 0.01 μg/L and a recovery of 102%.

### 2.3 Liquid‐chromatography tandem mass spectrometry (LC–MS/MS) analysis of approved acaricides in Israel

Honey samples were analyzed for trace levels of the approved acaricides coumaphos and amitraz following the method of Bommuraj *et al*.^20^ Briefly, 2 g of honey (collected from the in‐house beehive used for candy preparation) was mixed with 25 μL of a 10 mg/L internal standard solution (triphenylphosphate), 10 mL of double‐distilled water, and 10 mL of acetonitrile in a 50 mL centrifuge tube. After manual shaking to homogenize, a salt mixture (4 g anhydrous MgSO_4_, 1 g trisodium citrate dihydrate, 0.5 g disodium hydrogen citrate sesquihydrate, 1 g NaCl) was added. The mixture was vortexed for 2 min and centrifuged at 10 °C, 4500 rpm for 5 min. Subsequently, 6 mL of supernatant was transferred to a 15 mL tube containing 900 mg anhydrous MgSO_4_ and 150 mg PSA, vortexed for 30 s, and centrifuged again at 4500 rpm for 5 min. A 2 mL aliquot of the supernatant was evaporated to dryness under nitrogen at 40 °C, reconstituted in 1 mL methanol, diluted 1:2 with DDW, and subjected to LC–MS/MS analysis. Chromatographic separation was performed on an ACQUITY UPLC system (Waters Corp., Milford, MA, USA) with a XEVO TQD mass spectrometer, using a Zorbax SB‐C18 column (2.1 × 150 mm, 3.5 μm; Agilent Technologies) at 40 °C. Injection volume was 10 μL, with a flow rate of 0.4 mL/min. The mobile phase consisted of 10 mM ammonium acetate in water (A) and acetonitrile with 0.1% formic acid (B), using the following gradient: 95% A for 0.25 min, linear increase to 95% B over 0.25–7 min, held for 1 min, then returned to initial conditions by 10 min and held for 1 min. Positive electrospray ionization was used, and analytes were detected in multiple reaction monitoring (MRM) mode. Acaricide standards were prepared in water/acetonitrile (1:1) at 1 mg/L. Data acquisition and instrument control were performed using MassLynx software. LOQ values of coumaphos and amitraz were 0.005 and 0.01 mg/kg, respectively.

### 2.4 Candy formulation

Precisely 3.0 g of LiCl were weighed and thoroughly blended with 0.67 kg of sucrose powder using a 2 L polycarbonate laboratory blender (MRC, Harlow, UK). The final candy formulation was then prepared by mixing the LiCl‐sugar blend with 0.33 kg of honey, resulting in a final concentration of 50 mM LiCl in the candy. A control (blank) candy was prepared following the same procedure, omitting the addition of LiCl. The honey used for both formulations was confirmed to be free of Li residues and in Israel the only approved acaricides coumaphos and amitraz (see paragraph 2.2 and 2.3; Bommuraj *et al*. 2019).^20^ These sugar candy formulations served as the oral delivery method of LiCl for the experimental bee groups. A concentration of 50 mM was selected based on multiple studies demonstrating its efficacy in significantly reducing *Varroa* infestations when given in candy formulation while maintaining minimal toxicity to adult bees during a 10‐day exposure period.^9^, ^10^, ^11^, ^12^, ^13^, ^14^ Furthermore, we selected the candy formulation based on previous findings indicating its slower consumption rate over an extended period, which ensures a more continuous and gradual release of LiCl.^11^ This delivery profile offers a safer alternative compared to the syrup formulation.

### 2.5 Honey bees collected for *in vitro* behavioral assays

One‐day‐old bees were sourced from an apiary managed at the Volcani Institute, Israel, between June and August 2023. The bees used in all behavioral assays were local Italian strains commonly maintained in Israel and were not selectively bred for any specific behavioral traits. Broodcombs containing hatching workers were collected from the central area of the brood nest from two healthy colonies (one broodcomb per colony) to ensure uniform age and developmental stage of emerging bees. The broodcombs were transferred into a six‐frame hive box housed within a climate‐controlled chamber maintained at 34 ± 1 °C and 60 ± 5% relative humidity (RH) to facilitate bee emergence. After emergence, groups of newly emerged adult bees were placed into ventilated, transparent plastic cages for behavioral analysis as detailed below. All the *in‐vitro* behavioral experiments were performed at the Volcani Institute, The Department of Entomology, Israel.

### 2.6 Study design

#### 2.6.1 Effect of LiCl treatment on queen egg‐laying activity and *varroa* mortality

To examine the influence of LiCl on queen egg‐laying behavior, we used the assay described by Fine *et al*.^21^ In brief, 24 young (age of 3–4 weeks) egg‐laying queens were obtained from a commercial Israeli breeder (Noga Reuven, Prihey Hagalil, Israel). Each queen was placed in a queen monitoring cage with 50 1‐day‐old worker bees. The cages were provisioned with pollen cake made of 90% pollen, and 10% of a 60% sucrose solution in 5 mL tube, as well as 5 mL water tube, and 2 g of sugar candy *ad libitum*. The food was refreshed every other day. Half of the cages received 50 mM LiCl‐supplemented sugar candy, while the other half served as the control group with regular sugar candy. Egg‐laying plates were replaced daily, and the number of eggs laid was recorded each day from day three of the cage formation for 10 consecutive days. The observer was blinded to the treatment allocation during the experiment and measured daily egg‐laying rates in both the treatment and the control group. Queens that failed to lay eggs at all were excluded from the analysis. As only one such case occurred in the LiCl treatment group and two in the control, the final dataset included 11 cages in the LiCl group and 10 cages in the control group.

The acaricidal efficacy of 50 mM LiCl was assessed using the same cage setup described above. After a 10‐day exposure period, the total number of dead mites per cage was recorded for each treatment group. Varroa mites used in the assays were naturally associated with the 1‐day‐old worker bees collected from broodcomb, and no mites were artificially introduced into the experimental cages. As a result, the initial number of mites per cage was not standardized or quantified. Because the workers originated from the same source colonies, we assumed that mites were evenly distributed among cages. Although the total mite population per cage was not determined, the number of dead mites recorded at the end of the experiment provided valuable information by serving as an internal positive control for the activity of the lithium chloride treatment and by demonstrating its efficacy as an anti‐Varroa agent under the experimental conditions.

#### 2.6.2 Effect of LiCl treatment on worker nursing behavior

The effect of LiCl treatment on worker bee nursing behavior was assessed using the brood care assay as previously described by Shpigler and Robinson.^22^ Briefly, one‐day‐old worker bees were individually marked on their thoraces with a spot of paint (Testor's PLA, USA) with distinct colors and allocated into groups of 10 bees, which were maintained in vertically oriented Petri dishes (100 × 20 mm). Each Petri dish was provisioned *ad libitum* with pollen cake (composed of 90% pollen and 10% sugar water containing 50% sucrose), sugar candy, and water *ad libitum*. Experimental groups received sugar candy supplemented with LiCl at a concentration of 50 mM, while control groups were provided with sugar candy without LiCl supplementation. Each treatment consisted of 15 replicate dishes. One group of the LiCl treatment had fewer than seven bees after 7 days and was therefore excluded from subsequent analysis. After 7 days of treatment exposure, a 4‐day‐old queen larva in a queen cup was introduced into each Petri dish, and the bee behavior was monitored for a period of 5 min. Bee behavior was monitored continuously during a single 5‐min observation period for each group, and all visits to the queen cell and their duration were recorded. Each group was observed only once, as the bees used in the assays were naïve and repeated exposure is known to bias behavioral responses.^22^ To ensure robustness, 14–15 independent technical replicates were conducted for each treatment, providing a solid statistical basis for evaluating treatment effects.

Nursing behavior parameters recorded included: the number of bees attending the queen larva, the number of individual visits to the queen larva per cage, and the cumulative duration of visits per cage. Observations were conducted in a blinded manner to prevent observer bias regarding treatment allocation.

#### 2.6.3 Effect of LiCl treatment on worker aggression

Worker bee aggression following 50 mM LiCl treatment was evaluated using the intruder assay as previously described by Shpigler *et al*.^23^ Briefly, one‐day‐old worker bees were individually color‐marked and grouped in vertically oriented Petri dishes (100 × 20 mm), each containing 10 bees. Dishes were provisioned *ad libitum* with pollen cake (90% pollen, 10% sugar water with 50% sucrose), sugar candy, and water. Treatment groups received sugar candy supplemented with 50 mM LiCl, while control groups received unsupplemented sugar candy. Each treatment included 15 replicate dishes; one control and two LiCl dishes with fewer than seven bees after 7 days were excluded from analysis. On day 7, an intruder bee from a different freely foraging colony was introduced into each group for a single 5‐min observation period. Aggression behaviors (antennation, chasing, mandible opening, biting, and stinging) were scored from 1 to 5, reflecting increasing aggression intensity according to a previously established scoring system.^23^, ^24^ In the aggression assay, each group was tested once during a 5‐min observation period. The aggressive behaviors were recorded, and behaviors lasting longer than 10 s (e.g., biting) were rescored at 10‐s intervals according to the established protocol.^23^, ^24^ The intruder bee was not removed after the observation period, as the group was not used for any subsequent tests, ensuring that repeated exposure did not bias the behavioral responses. The following metrics were analyzed per treatment: mean number of highly aggressive bees (individual score ≥ 10), mean highest individual aggression score per group, and mean additive aggression score per group. Observations were conducted blind to treatment allocation.

### 2.7 Statistics

Linear mixed‐effects model was applied to evaluate differences in the daily egg‐laying rates between LiCl treatment and the control group as a function of time. The LiCl treatment group consisted of 15 individual queen bees, while the control group comprised 15 individual queen bees. Due to the repeated measures design, with egg‐laying monitored over consecutive days for each queen bee, a mixed‐effects model was applied, accounting for both fixed effects (treatment, time, and their interaction) and random effects (individual queen bee). The Geisser–Greenhouse correction was utilized to adjust for potential deviations from the sphericity assumption. Post‐hoc multiple comparisons between treatment groups at individual time points were corrected using the Sidak method. Statistical significance was set at α = 0.05. The statistical analyses were conducted using GraphPad Prism software (GraphPad Software, Inc. 10.4.1).

Due to failure of normality and log‐normality tests (D'Agostino & Pearson test, Anderson‐Darling test, Shapiro–Wilk test, Kolmogorov–Smirnov test), anti‐Varroa activity of 50 mM LiCl *vs* control group, worker nursing behaviors as well as worker's aggression behavior between control and LiCl‐treated groups were compared using the non‐parametric two‐tailed Mann–Whitney *U* test with a significance threshold set at *α* = 0.05. Statistical analyses were performed using GraphPad Prism software (version 10.4.1; GraphPad Software, Inc.).

## 3 RESULTS

### 3.1 Determination of Li and acaricide residues in blank honey

The concentration of Li in the blank honey sample used for sugar candy preparation was determined to be 2.4 μM, which is over 2000‐fold lower than the spiked LiCl concentration of 50 mM. This finding aligns with previously reported background Li levels in honey samples in the range of 10–120 μM confirming that the intrinsic Li concentration in the honey is negligible relative to the experimental LiCl spike.^10^, ^11^, ^13^, ^17^, ^25^ Additionally, residues of amitraz and coumaphos were below detection limits in the tested honey. Therefore, observed behavioral effects in this study can be confidently attributed to the experimentally introduced 50 mM LiCl.

### 3.2 Effect of 50 mM LiCl treatment on queen egg‐laying activity and on *varroa* mortality

**Figure 1:**
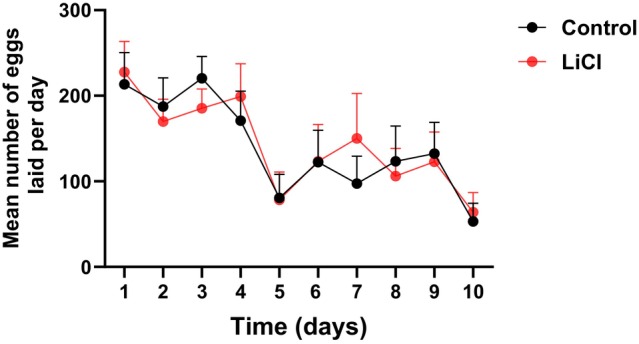
Effect of 50 mM LiCl treatment on daily queens' egg laying activity. The mean number of eggs in each day was compared between control queens (black, *n* = 10) to LiCl treated queens (red, *n* = 11) for 10 days. Statistical analysis was performed using linear mixed‐effects model, with a significance threshold of *α* = 0.05.

LiCl treatment did not affect the queen's egg‐laying rate in the laboratory‐based queen monitoring cages over the 10‐day treatment period (Fig. 1). No statistically significant differences between the LiCl‐treated queens (*n* = 11) and the control queens (*n* = 10) were observed according to the generalized linear mixed‐effects model (*F*_(1,17)_ = 0.3, *P* = 0.86; z_(1,17)_ =0.78, *P* = 0.44). The rate, daily number of laid eggs, and total egg production over 10 days were similar between the two groups.

**Figure 2:**
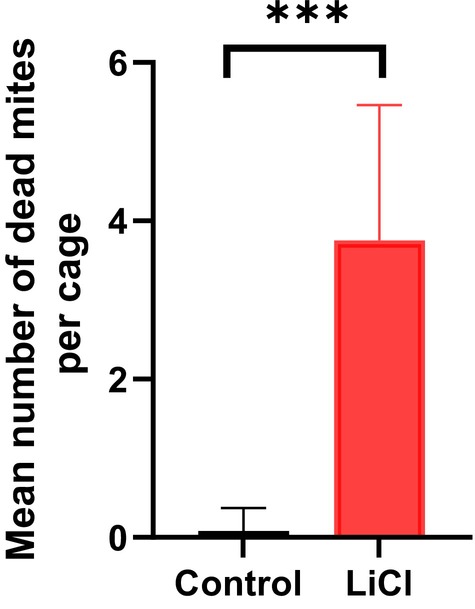
Anti‐Varroa efficacy of 50 mM LiCl as compared to control group. The mean number of dead *Varroa* mites per treatment group was determined after 10 days of daily exposure of worker bees (*n* = 50 bees per cage with 12 cages in the control group and 12 cages in the treatment group) to sugar candy spiked with LiCl or blank sugar candy. Statistical analysis was performed using the two‐tailed Mann–Whitney *U* test, with a significance threshold of α = 0.05. ***, *P* < 0.0001.

As shown in Fig. 2, worker bees exposed to 50 mM LiCl for 10 consecutive days exhibited a significant increase in mite mortality as compared to the control group (*P* < 0.0001). Notably, worker bee mortality was negligible and not different in either group, indicating that the 50 mM LiCl dosage in the candy formulation is well tolerated to adult worker bees (data not shown). These findings are consistent with previous studies reporting the efficacy and safety of LiCl as a varroacide in treating phoretic *Varroa* infestations in worker bees.^10^, ^11^

### 3.3 The effect of LiCl treatment on worker nursing behavior

**Figure 3:**
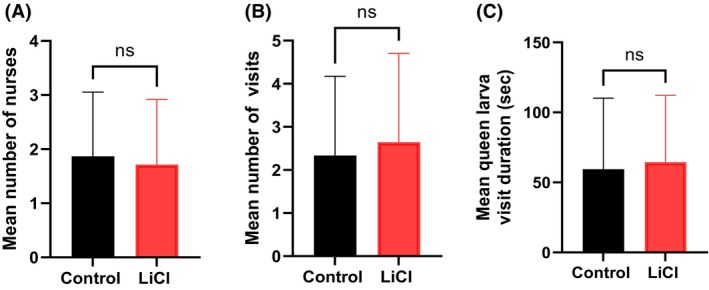
Effects of LiCl treatment on nursing behavior in worker honey bees. Worker bees were exposed to a single intruder bee for a duration of 5 min. The control group (*n* = 15) and the LiCl‐treated group (*n* = 14) were compared. (A) Effect of LiCl treatment on the number of nursing bees attending the queen larva. (B) Frequency of visits by nursing bees to the queen larva. (C) Mean duration (in sec) of visits by nursing bees during a 5‐min observation. Statistical analysis was performed using the two‐tailed Mann–Whitney *U* test, with a significance threshold of *α* = 0.05. NS indicates no statistically significant difference.

In the brood care assay, LiCl treatment did not influence the bees' nursing behavior under laboratory conditions. The average number of nursing bees was comparable between treatments, with the LiCl group containing 14 and the control group containing 15 cages (*P* = 0.78; Fig. 3(A)). Similarly, there were no significant differences in the number of visits to the queen cell (*P* = 0.67; Fig. 3(B)) or in the total duration of the treatment time of the bees to the larvae (*P* = 0.71; Fig. 3(C)).

### 3.4 The effect of LiCl treatment on worker's aggression

**Figure 4:**
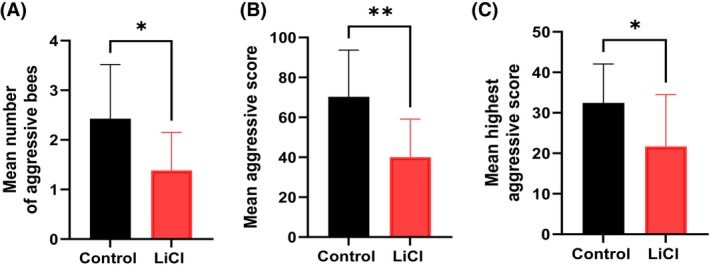
Effects of LiCl treatment on aggressive behavior in worker honey bees. Worker bees were exposed to a single intruder bee for a duration of 5 min. The control group (*n* = 14) and the LiCl‐treated group (*n* = 13) were compared. (A) Mean number of aggressive workers per group. (B) Mean cumulative aggression score directed toward the intruder bee. (C) Mean maximum aggression score recorded for an individual worker in each group. Asterisks denote statistically significant differences: (A) *P* = 0.012; (B) *P* = 0.0011; (C) *P* = 0.045.

LiCl treatment significantly reduced worker aggression toward intruder bees in the laboratory assay (Fig. 4). The mean number of highly aggressive bees (aggressive score ≥ 10) in the LiCl‐treated group was 1.7‐fold lower than in the control group (*P* = 0.008; Fig. 4(A)). The additive aggression score of the group was significantly lower in LiCl‐treated bees (mean = 40.0) compared to controls (mean = 70.2; *P* = 0.001; Fig. 4(B)). Additionally, the maximum individual aggression scores within LiCl‐treated groups were significantly lower than those in control groups (*P* = 0.027; Fig. 4(C)). These findings demonstrate that LiCl treatment effectively reduces bee aggression at both individual and group levels.

## 4 DISCUSSION

LiCl has emerged as a promising treatment for *Varroa* infestations. Although its efficacy in mite control and low acute toxicity to worker bees have been well documented its potential effects on queen bee health—including reproductive performance—as well as on worker bee nursing behavior and overall aggression, remain unexplored to date.^10^, ^11^, ^14^

The significant increase in *Varroa* mortality following continuous exposure of worker bees to 50 mM LiCl in the form of candy over a 10‐day period (Fig. 1) align with previous reports by Rein *et al*. (2022 & 2024), which highlighted the efficacy of LiCl in reducing phoretic mite loads without adversely affecting worker bee survival.^10^, ^11^ The observed safety profile further reinforces the suitability of LiCl for field applications, particularly when administered in formulations that enable gradual release such as candy formulations. This contrast with earlier methods involving syrup‐based delivery, which have been associated with higher risks of toxicity, especially under prolonged or repeated exposure.^12^, ^13^, ^14^

Nevertheless, concerns regarding potential long‐term effects of LiCl exposure on honey bees, especially their brood and contamination of hive products remain a critical issue.^10^, ^11^, ^17^ Prešern *et al*. demonstrated that treatment with 25 mM LiCl resulted in detectable Li residues in honey and bee tissues, highlighting potential risks associated with prolonged use in apicultural practices.^17^

Rein *et al*. recently highlighted potential hazards associated with LiCl treatments by evaluating different application strategies for Varroa control, with a particular focus on brood development.^11^ Substantial differences in brood survival were observed depending on dosage, timing, frequency, and exposure duration. A regimen of 2.5 kg of 50 mM LiCl‐spiked candy administered over 9 days resulted in severe brood toxicity, with only 4.7% survival recorded on day 16. In contrast, administration of 2 kg over 5 days yielded 45% survival at day 3, after which the colony gradually recovered, reaching 82% brood survival by day 19. A third regimen (0.5 kg every 7 days across four treatments) initially showed 52% survival, but this dropped sharply to 8% after the third application, reflecting cumulative toxicity. These findings underscore the importance of optimizing LiCl treatment protocols to achieve effective Varroa control while minimizing brood loss. The adverse effects of LiCl on honey bee brood reported by Rein *et al*. may result either from direct toxicity to the developing larvae and/or from alterations in worker nursing behavior.^11^ Impairments in nursing behavior could compromise the care provided to the larvae, thereby reducing their chances of survival. In the present study, we assessed the impact of LiCl treatment on honey bee nursing behavior using the brood care assay. The lack of significant differences in nursing activity between the LiCl‐treated and control groups supports our hypothesis that LiCl toxicity primarily affects developing larvae rather than the caregiving behavior of worker bees. This finding is consistent with the observations of Rein *et al*., who reported that oral administration of 50 mM LiCl did not adversely influence subsequent colony growth or overwintering success, despite initial brood removal and the continued presence of the same workers within the colony.^11^ These findings suggest that while LiCl may induce temporary brood disturbances, it does not compromise essential worker behaviors such as nursing. Notwithstanding, it is important to note that our study was conducted under laboratory conditions, which may not fully replicate the complexities of a natural hive environment. Factors such as colony size, environmental stressors, and the presence of pathogens could influence the effects of LiCl on bee behavior and colony dynamics. Therefore, while our findings provide valuable insights into the specific aspect of nursing behavior, further field studies are warranted to comprehensively assess the safety and implications of LiCl use in apiculture.

Furthermore, we have demonstrated that LiCl treatment significantly mitigated aggressive behaviors at both individual and group levels (Fig. 4). To our knowledge, this is the first study to report the impact of LiCl on honey bee aggression. This aligns with existing literature on the neuromodulatory effects of Li in other species, where it has been shown to influence behaviors such as aggression and mood regulation.^26^, ^27^ The mechanism by which Li reduces aggression in honey bees remains to be elucidated. In mammals, Li is known to modulate neurotransmitter systems, including serotonin, dopamine and inositol pathways, which play roles in aggression and mood disorders.^28^ It is plausible that a similar mechanism exists in honey bees, where Li influences neural circuits governing aggression. Further neurobiological studies are required to investigate this hypothesis.

It is important to consider the broader implications of reduced aggression in honey bee colonies. Aggressive behavior serves as a defense mechanism against predators and intruders; thus, a diminution could potentially affect colony defense.^29^ However, excessive aggression can also lead to increased colony stress and may impact foraging efficiency and colony cohesion. Therefore, the modulation of aggression by Li could have complex effects on colony dynamics, warranting comprehensive field studies to assess the net impact on colony health, foraging behavior and productivity.

This study confirms that LiCl effectively reduces *Varroa destructor* infestations without impairing queen egg laying or worker nursing behavior. While previous studies have raised concerns about brood toxicity and residue accumulation, our results suggest that such effects are linked primarily to direct larval sensitivity rather than changes in caregiving behavior. A novel finding is that LiCl significantly reduces worker aggression, consistent with its known neuromodulatory effects in other species. Reduced aggression may lessen colony stress but could also affect colony defense, underscoring the need for further field studies.

## 5 CONCLUSIONS

This study addresses critical gaps regarding the behavioral impacts of LiCl treatment on honey bees, specifically focusing on queen egg laying, workers nursing and aggressive behaviors. LiCl administered at 50 mM did not adversely influence queen egg laying or workers' nursing behavior, reinforcing previous findings suggesting negligible long‐term risks to essential colony maintenance activities. Conversely, LiCl significantly reduced aggression among worker bees at both individual and group levels—a previously unreported effect in honey bees, consistent with Li's established neuromodulatory role in mammals. This reduction in aggression may offer potential benefits for managing colony stress, especially with aggressive strains like the Africanized honey bees.^30^ However, the ecological and adaptive consequences for colony defense require careful consideration.

Despite LiCl's proven efficacy against *Varroa* mites and low acute toxicity to adult bees, concerns persist regarding chronic effects on brood development and residue accumulation in hive products. Adjustments in dosage and duration of LiCl exposure may mitigate these risks, emphasizing the importance of carefully tailored treatment protocols. Further neurobiological studies should elucidate the mechanisms underlying LiCl's modulation of aggression. Ultimately, comprehensive field trials are necessary to fully assess LiCl's practical implications, balancing varroacidal efficacy against potential behavioral and developmental risks to ensure sustainable apicultural practices.

## AUTHOR CONTRIBUTIONS

SS conducted the experiment and collected the data, OA and SZ worked with the bees in the field, HYS and JS conceptualize the study and the experiment design, analyzed the data and write the manuscript.

## DISCLOSURE

The graphical abstract was generated using *ChatGPT‐5* (OpenAI, San Francisco, CA, USA) based on the authors’ original data and conceptual design. The authors reviewed and verified the graphical abstract and assume full responsibility for its accuracy and integrity.

## FUNDING INFORMATION

The Israeli Pollination and Honey Council.

## CONFLICT OF INTEREST

The authors have no relevant financial or non‐financial interests to disclose.

## Data Availability

The data that support the findings of this study are available on request from the corresponding author. The data are not publicly available due to privacy or ethical restrictions.
